# Birch allergen challenges in allergic conjunctivitis using standard conjunctival allergen challenge and environmental exposure chamber

**DOI:** 10.1002/clt2.12053

**Published:** 2021-08-17

**Authors:** Alina Gherasim, Jean‐Luc Fauquert, Nathalie Domis, Xavier Siomboing, Frederic de Blay

**Affiliations:** ^1^ ALYATEC Environmental Exposure Chamber Strasbourg France; ^2^ University Hospital Clermont‐Ferrand France; ^3^ Soladis Clinical Studies Roubaix France; ^4^ Chest Diseases Department Strasbourg University Hospital Strasbourg France; ^5^ University of Strasbourg Strasbourg France

**Keywords:** birch allergy, conjunctival allergen challenge, conjunctivitis, environmental exposure chamber

## Abstract

**Background:**

Environmental exposure chambers (EECs) have been used extensively to study allergic rhinoconjunctivitis. Few studies have been published using EECs in conjunctivitis only, and none have used conjunctival allergen challenge as a selection criterion. The present study validated ALYATEC EEC in allergic conjunctivitis to birch pollen.

**Methods:**

Sixteen patients with a positive conjunctival allergen challenge (CAC) were exposed to 60 ng/m^3^ of Bet v 1 in an EEC on two consecutive days for a maximum of 4 h to validate EEC exposure to birch. Reproducibility was tested among seven of the patients. A conjunctival positive scoring during the CAC and the EEC exposure was defined as a Total Ocular Symptom Score (TOSS) ≥ 5.

**Results:**

Fifty percent of patients had a conjunctival positive scoring during first exposure and 75% during second exposure. The mean time to a conjunctival response was 81.2 ± 33.9 min and 101.6 ± 57 (*P* > 0.05) during first and second exposure, respectively. No difference in TOSS occurred between the two exposures. The time necessary to obtain a positive response during the CAC was significantly shorter than with the EEC. The estimated quantity of Bet v 1 inducing a positive response was 0.07 ± 0.03 ng (exposure 1), 0.07 ± 0.07 ng (exposure 2), 980 ± 784 ng (CAC). Conjunctival positive scoring and quantity of Bet v 1 was reproducible in all six EEC exposures.

**Conclusions:**

Early conjunctival responses induced by birch allergen exposures in EEC were different than from those identified with direct instillation during CAC. EEC appears to be closer to natural exposure than CAC.

## 1 BACKGROUND

Allergic conjunctivitis occurs in atopic individuals exposed to specific antigens and manifests as an early reaction, within minutes or hours of exposure to allergens, and may or may not be associated with allergic rhinitis.^1^ IgE‐mediated hypersensitivity inducing conjunctivitis is frequent, from 15% to 20% in general practice^2^, ^3^ to 40% in the US population when examined in an ophthalmological survey.^4^

In France, the prevalence of seasonal allergic rhinoconjunctivitis (SAR) was found to be 13% among 10‐year‐old children and approximately 20% among adults.^5^, ^6^ Birch is one of the most frequent sources of allergens that induce rhinoconjunctivitis.^7^ In the general French adult population, the prevalence of birch sensitization was 4.7%.^8^ In a recent analysis, a statistically significant correlation has been found between the birch pollen concentration levels during the pollen season as well as the peak pollen period and the pollen‐induced symptoms such as Total Nasal Symptom and Medication Score (TNSMS).^9^ In another study, the frequency of the ocular response to natural exposure to birch pollen in sensitized participants followed a liner progression until birch daily average concentrations reached a plateau of 110 grains/m^3^, with a cutoff of ocular symptoms at 70 grains/m^3^ at the beginning of the season.^5^

In 1990, Abelson et al. demonstrated a correlation between skin sensitization (grass pollen, ragweed pollen, and cat allergen) and positivity to conjunctival allergen challenge (CAC). This tool confirms allergen involvement in the diagnosis of allergic conjunctivitis, allowing precise selection of the participants in clinical studies. The CAC model is the only clinically validated method recognized by Food and Drug Administration (FDA) for testing the efficacy of eye anti‐allergic molecules.^10^ The practical aspects were described in a position paper of the European Academy of Allergy and Clinical Immunology (EAACI) Task Force.^11^

Environmental exposure chambers (EECs) have been in development since 1985 to study new therapeutics for allergic pathologies, including conjunctivitis.^12^, ^13^ EECs have the advantage of achieving reproducible and safe exposure with controlled levels of allergen for several hours in several subjects simultaneously by avoiding confounding factors during exposure.^14^, ^15^ The ALYATEC EEC has been validated in mite and cat‐induced allergic asthma.^16^, ^17^ These studies demonstrated that the allergen exposures are standardized with an inter‐test coefficient of variation of less than 30%.

To validate the ALYATEC EEC with birch pollen, we exposed patients affected by seasonal allergic conjunctivitis caused by birch pollen to airborne birch allergen. We investigated the amount of Bet v 1 (*Betula verrucose* major allergen) and the time necessary to induce a conjunctival response in at least 50% of participants evaluated on TOSS scoring of at least 5 (primary endpoint). We also evaluated the reproducibility of the EEC method.

## 2 METHODS

### 2.1 Demographic characteristics

Participants aged from 18 to 65 years were selected for eligibility based on having a history of more than two years of moderate allergic conjunctivitis during birch pollen season.^1^, ^11^ Allergic sensitizations were documented by a positive skin prick test to birch allergen with a wheal diameter ≥6 mm compared to negative control and positive birch‐specific IgE (>0.10 kU/l). The main inclusion criterion was a conjunctival positive scoring (defined as TOSS scoring reaching up to five points 15 min after instillation of the culprit allergen) during an individual CAC.^10^, ^11^ The study was performed outside the pollen season (September–December) in France. A 7‐days washout period was required for topical or systemic anti‐histamines or other ophthalmic treatment. Exclusion criteria were evaluated prior to inclusion and were as follows: participants experiencing moderate ocular symptoms in the previous week; participants who received systemic long‐acting corticosteroids within the past 4 weeks; ocular laser treatment within the past 3 months; ocular surgery within the last 6 months; abnormality or clinically significant current ocular disorder, including symptoms of allergic conjunctivitis; ongoing immunotherapy to any allergen or, within the last 5 years, to birch allergen.

### 2.2 Interventions

**FIGURE 1:**
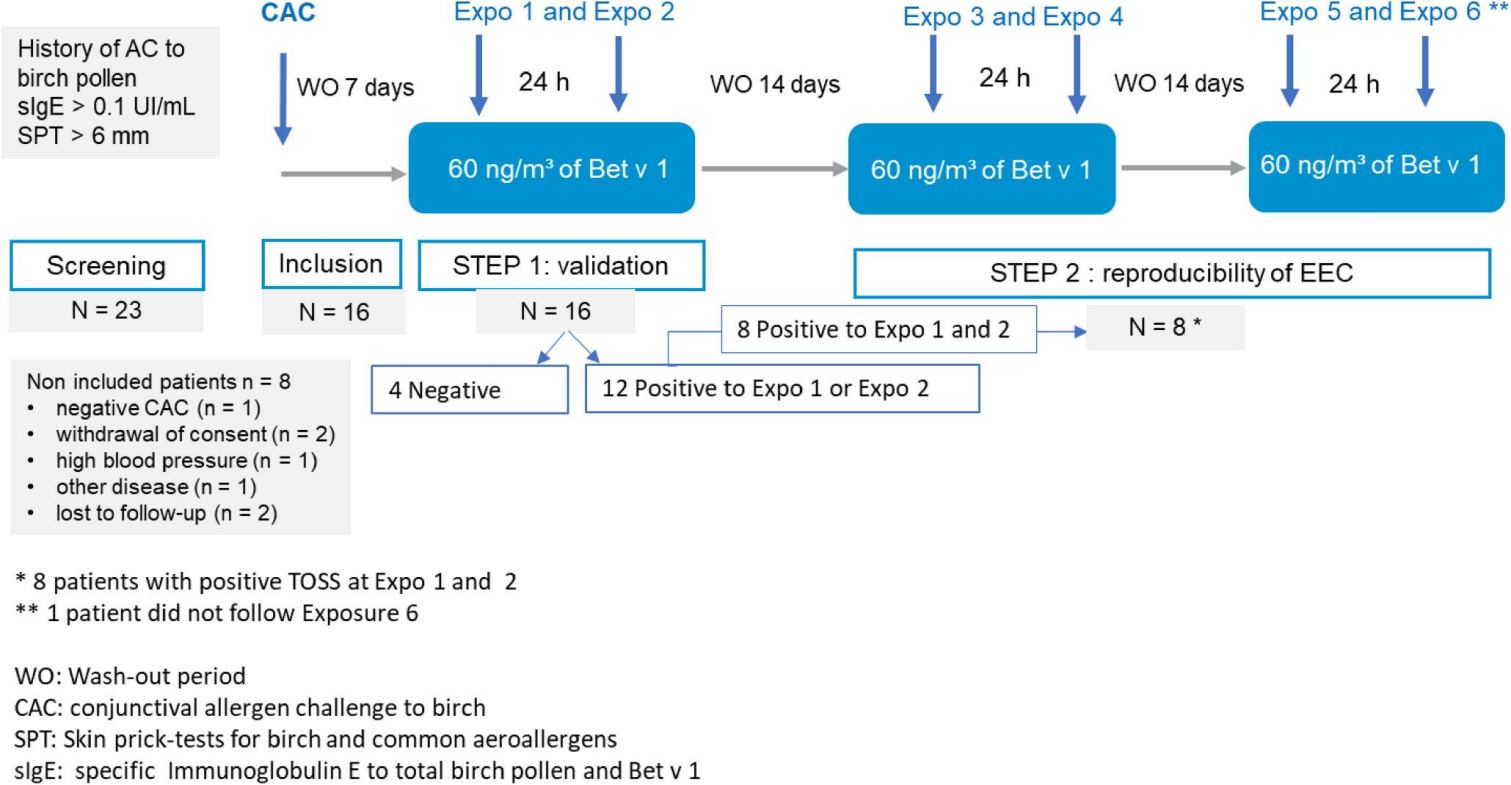
Study design

This was an open, single‐center study designed to determine the concentration of airborne Bet v 1 inducing an allergic conjunctivitis response in participants allergic to birch pollen during allergen exposures to birch pollen extracts in the EEC. During the first screening visit, the patient gave informed written consent and underwent the following procedures and assessments: medical history review, skin prick testing to birch pollen allergen (ALK*‐Abello®*), and a blood draw for investigating specific IgE to birch (*Betula verrucose,* Phadia ImmunoCap, Thermofisher®). The second screening visit was for an individual CAC to birch allergen. All responders in the CAC were included in the present study (Figure 1).

The CAC was performed according to the updated EAACI guidelines.^1^, ^11^ This procedure consisted of the instillation of 20 µl of diluted birch allergen extract (100 IR lyophilized extract, Stallergenes Greer®) in the inferior‐external quadrant of the bulbar conjunctiva in incremented dilutions at 10‐min intervals: 3, 6, 12, 25, 50, and 100 IR/ml.^11^ The clinical response was assessed by the Total Ocular Symptom Score (TOSS) with the same cumulated positivity criteria for the CAC and EEC exposure. If the TOSS was <5 at 10 min after each instillation, the test was considered negative. The next concentrated dose was then instilled until a positive response was reached. One week after CAC, patients were enrolled for EEC visits.

Step 1 of the study consisted of two EEC exposures in consecutive days to the same birch pollen extract (Expo 1 and Expo 2). This was sustained to mimic daily life, and to reproduce an eventual priming effect. The primary endpoint of the study was met when at least 50% of the participants had a conjunctival positive scoring. The main judgment criteria were the amount of Bet v 1 inducing a conjunctival response after EEC exposure. In addition, we compared the intensity of the clinical response induced in participants exposed to birch pollens using the TOSS and the mean time to reach a positive ocular response (i.e., TOSS ≥ 5). In step 2, we studied the reproducibility of the allergen exposure. Participants who responded to Expo 1 and Expo 2 were enrolled in step 2 and exposed two additional times on two consecutive days (from Expo 3 to Expo 6). Each double EEC exposure test was separated by 14 days.

### 2.3 Clinical assessments

The TOSS was used during both the CAC and EEC exposures. This score was first described^10^ as the sum of four conjunctival symptom scores: itching, redness, chemosis, and tearing (range: 0–13). It was evaluated after instillation of the allergen on one side, with the other eye serving as a negative control after instillation of the NaCl solution**.** A slit lamp examination was used to score redness and chemosis only. Itching was assessed by the patient using a 5‐point severity scale from 0 (none) to 4 (very severe: incapacitating itch with irresistible urge to rub), with 1 = mild (intermittent tickling sensation), 2 = moderate (continual awareness but without the desire to rub), and 3 = severe (continual awareness with the desire to rub). For ocular redness, ratings were collected for the nasal and temporal area of each eye and averaged by the study physician using a four‐point severity score (0 = absent; 1 = mild: perhaps localized within some quadrant, 2 = moderate: more marked and diffuse reddening in the quadrants; 3 = severe: very marked and diffuse reddening in the quadrants). Tearing was also rated by the physician using a 4‐point severity score (0 = absent; 1 = mild, eyes feel slightly watery; 2 = moderate, blows nose occasionally; and 3 = severe, tears rolling down cheeks). Chemosis was rated by the study physician as follows: 0 = absent, 1 = mild (detectable with slit lamp, conjunctiva separated from sclera), 2 = moderate (visually evident, raised conjunctiva, especially at the limbal area), and 3 = severe (ballooning of conjunctiva). The patient left the EEC when the mean TOSS of both eyes was ≥5 (Table 1).

**TABLE 1:** Scoring system to measure the signs and symptoms of allergic conjuntivitis. Data from Abelson et al. Arch Ophthamol 1990;18:84

Redness	Chemosis	Tearing	Itching (to Be graded by subject)
0 = none	0 = none	0 = none	0 = none
1 = mild (perhaps localized within some quadrant)	1 = mild (detectable with slit lamp, conjunctiva separated from sclera)	1 = mild tearing (eyes feel slightly watery)	1 = mild (intermittent ticking sensation)
2 = moderate (more marked and diffuse reddening in the quadrants)	2 = moderate (visually evident, raised conjunctiva, especially at the limbal area)	2 = moderate tearing (blows nose occasionally)	2 = moderate (continual awareness, but without the desire to rub)
3 = severe (very marked and diffuse reddening in the quadrants)	3 = severe (ballooning of conjunctiva)	3 = severe tearing (tears rolling down cheeks)	3 = severe (continual awareness with the desire to rub the eyes)
			4 = incapacitating itching (subject insist on rubbing eyes)

*Note*: TOSS was perforemd in both CAC and EEC. Regarding rednees and chemosis, the sum of scores obtained for both eyes was calculated during EEC exposures.

Safety monitoring was performed by clinical survey and pulmonary function assessment. The Total Nasal Symptom Score (TNSS) and portable spirometry were performed every 20 min during exposure. Early asthma response was defined as a drop in FEV 1 of 20% (Forced expiratory volume in 1 s), leading to disruption of the ongoing EEC exposure**.**

At the end of an exposure, all participants were treated as needed with topical antihistamines, eye drops, and oral second generation H1‐antihistamines according to the persistence and severity of the conjunctival or rhinitis symptoms. When an early asthmatic response occurred, participants remained under supervision for 6 h. Thereafter, they were discharged with a rescue therapy kit containing oral antihistamines, topical mast cell stabilizers, and short‐acting beta 2 agonist inhaler.

This study was approved by an independent ethics committee and was conducted according to Good Clinical Practice (GCP) standards using the guidance documents and practices offered by the International Conference on Harmonization (ICH) and European directive 2001/20/CE. The study was registered at ClinicalTrials.gov under number NCT04641130.

### 2.4 Environmental exposure chamber

ALYATEC EEC is a new generation EEC located in Strasbourg, France. It was conceived as a clean room with low‐emitting, non‐adherent and easily‐cleaned materials. The absence of endotoxins and VOCs levels is periodically evaluated. The volume of the EEC is 147 m^3,^ (area = 65 m^2^) with a capacity up to 20 seats. During allergen exposures, airborne particles number and size are continuously monitored in order to ensure homogenous distribution of allergen^16^, ^17^ In this study, participants were exposed to the same batch of lyophilized birch allergen extract (100 IR; *Stallergenes Greer*®), diluted in a NaCl solution, as the one used for the CAC. Before each EEC exposure, lyophilized birch allergen was prepared in a NaCl solution and nebulized in the EEC. The concentration of Bet v 1 in the allergen extract was measured before and after nebulization, using ELISA. Nebulization of birch allergen was initiated 30 min before patients entered the EEC, allowing to reach a stable plateau of airborne particles. The homogeneous distribution of allergen was ensured by using 10 particle counters in the EEC with real‐time monitoring. Therefore, the size and number of particles were continuously monitored during 4 h of exposure.

Online measurements of the temperature, relative humidity, and air exchange were provided as described in the previous studies.^16^, ^17^ After each allergen exposure, allergen was collected on five glass fiber filters located next to the participants' chairs during exposure to determine the Bet v 1 concentration using ELISA (Indoor Biotechnologies®). The concentration of the Bet v 1 airborne allergen was estimated to be 60 ng/m^3^. During each EEC allergen exposure, the conjunctival scoring was assessed every 10 min during the first hour and then every 20 min.

### 2.5 Quantity of Bet v 1 (Q) inducing a conjunctival positive scoring

As the allergen affected the ocular surface, we used the tear film renewal rate (TRR) to estimate the amount of extract applied during EEC exposure. The TRR was calculated to be 154 × 10^6^ mm^3^/min by Beaudouin et al.^18^ Thus, the quantity (Q) of extract applied was calculated in nanograms as follows: *Q* = C x Time x TRR, where C is the concentration of airborne Bet v 1 in ng/m^3^ and Time is the time required to induce the conjunctival positive scoring in minutes.

### 2.6 Statistical methods

All statistical analyses were performed using SAS^®^ guide Enterprise software (SAS Institute). Missing data were not replaced. Continuous variables were described as the number of observed data, mean, and standard deviation of normally distributed values, or median (interquartile range)**.** Categorical variables were described as the participants' size and percentage in each category. For inferential statistics, *P*‐values < 0.05 were considered significant. Tests were two‐tailed. Reproducibility was tested using a Pearson correlation between exposure days.

## 3 RESULTS

### 3.1 Participants

Among 23 screened participants, 16 met the inclusion criteria and performed two consecutive EEC exposures. Eight participants who responded at Expo 2 were included in step 2 (Figure 1). Patient characteristics are given in Table 2. Roughly, sensitization to birch pollen was evidenced by the skin prick test mean wheal diameter of 7.4 ± 1.6 mm and mean specific IgE value of 61.3 ± 109.3 kUI/L for the total study population. Moreover, the median of birch specific IgE levels was comparable in the two steps with 20.7 kU/L in the STEP 1 and 15  kU/L in the STEP 2, respectively. Co‐sensitizations were frequent, as only one patient was mono‐sensitized to birch pollen. At inclusion, 15 of the 16 included patients were affected by birch pollen rhinitis (7 with mild and 8 with moderate‐to‐severe rhinitis). Furthermore, 9 out of 16 participants presented with asthma according to GINA (Global Initiative for Asthma) class

**TABLE 2:** Study population

Data mean ± standard deviation or *n* (%)	STEP 1	STEP 2
Number of participants	16	8
Age, years	26.4 ± 6.8	26.6 ± 7.5
Gender (male)	10 (62.5%)	5 (62.5%)
Skin prick test to birch, mm	7.4 ± 1.6	7.7 ± 1.6
Birch specific IgE, kU/I	61.3 ± 109.3	54.6 ± 114.1
Mono‐sensitized to birchSensitized to common aeroallergens^a^	1/16 (6.2%) †	0/8 (0%)
	15/16 (93.7%) ‡	8/8 (100%)
Asthmatic participants	9/16 (57%)	4/8 (50%)
Rhinitis to birch, *n* (%) MildModerate to severe	15/16 (93.7%)7/16 (43.7%)8/16 (50%)	3/8 (37.5%)2/8 (25%)1/8 (12.5%)
Mean predictive FEV1, %	103.7 ± 9.6	101.63 (7.1)
CAC provocative dose of Bet v 1, ng	507.5 ± 392.2	385 ± 349
CAC cumulative dose of Bet v 1, ng	980.0 ± 784.5	735.0 ± 698.0

^a^ Co‐sensitization was frequent with grass and ash pollen in 87.5 % and 56.2 % of patients respectively.

**TABLE 3:** Conjunctival responses during CAC and EEC (step 1)

Positivity threshold TOSS ≥ 5	CAC *n* = 16	EEC Day 1 *n* = 8^a^	EEC Day 2 *n* = 12^b^
TOSS units	6.2 ± 1.1	5.7 ± 0.8 n.s.	5.5 ± 0.6 n.s.
TOSS [min; max]	[0; 9]	[0; 7]	[0; 7]
Time until positivity, min	35 ± 15.1	81.2 ± 33.9*	101.6 ± 57.3*
Cumulative dose of birch allergen exposure, ng	980 ± 784.50	0.07 ± 0.03**	0.07 ± 0.07**

*Note*: Data are given as mean ± standard deviation. Step 1 (EEC and CAC) *n* = 16. Comparison with CAC (Pearson test):* Time *p* < 0.05; **Cumulative Dose *p* < 0.001.

Abbreviations: EEC, environmental exposure chamber; CAC, conjunctival allergen challenge; TOSS, Total Ocular Symptom Score; n.s., not significant.

^a^ Exposure 1: 8 out of 16 participants.

^b^ Exposure 2: 12 out of 16 participants had positive TOSS.

### 3.2 Conjunctival outcomes during CAC and EEC exposures (step 1) (Table 3)

Among the 16 participants included in step 1, 8 patients (i.e., 50%) had a conjunctival positive scoring during the first exposure and 12 patients (i.e., 75%) during the second one. The mean TOSS was 5.7 ± 0.8 after Expo 1 and 5.5 ± 0.6 after Expo 2, whereas the mean TOSS after CAC was 6.2 ± 1.1. No correlation was observed between the TOSS in the EEC versus CAC (*r* = 0.05). The maximal TOSS for both types of exposure was 9 after CAC and 7 after EEC exposure. The mean time needed to obtain a positive conjunctival challenge was not significantly different between Expo 1 (81.2 ± 33.9 min) and Expo 2 (101.6 ± 57 min). During the CAC, positivity was obtained in 35 ± 15 min. The estimated quantity of Bet v 1 inducing a conjunctival positive scoring was 980 ng in the CAC and 0.07 ± 0.03 ng during Expo 1 and 0.07 ± 0.07 ng during Expo 2. This level was significantly lower with the EEC than the CAC (Table 3). No nasal response was observed during and after CAC.

### 3.3 Reproducibility of EEC exposures (step 2)

The eight participants included in step 2 had identical characteristics to the whole cohort from step 1 (Table 2). One patient dropped out of the study before the last exposure and was not analyzed here. This patient left the EEC before reaching a positive TOSS due to an early asthma response. Among the seven remaining participants, all except one exhibited a conjunctival positive scoring to the entire course of six designed exposures (Expo 1 to Expo 6; Table 3). The clinical response was identical throughout the six exposures in terms of TOSS. Moreover, the time necessary to reach TOSS ≥ 5 was <2 h for all exposures. The amount of Bet v 1 inducing a conjunctival positive scoring was similar in all six exposures. Reproducibility was studied regarding the time and quantity of allergen necessary for a positive challenge. Time of exposure was highly reproducible (Table 4) with a Pearson correlation coefficient of 0.78 (*p* < 0.05) between Expo 2 and Expo 4. As for the quantity of allergen inducing a positive response, reproducibility was also assessed between Expo 2 and Expo 4 with a Pearson correlation coefficient 0.81 (*p* = 0.028).

**TABLE 4:** Reproducibility of results with EEC exposure in seven participants (step 2)

Positivity threshold TOSS ≥ 5	Expo 1	Expo 2	Expo 3	Expo 4	Expo 5	Expo 6
TOSS Scoring units	5.7 ± 0.8	5.5 ± 0.6	5.8 ± 1.2	5.7 ± 1.1	6.8 ± 1.5	6.3 ± 1.9
TOSS Scoring units [min; max]	[0; 7]	[0; 7]	[0; 8]	[0; 8]	[0; 9]	[0; 9]
Time until positivity, min	68.3 ± 25.6	91.4 ± 62.03*	77.1 ± 34.9	82.8 ± 31.4 *	72.8 ± 28.1	65.7 ± 21.4
Cumulative dose of birch allergen exposure, ng	0.06 ± 0.02	0.08 ± 0.06**	0.07 ± 0.03	0.08 ± 0.03**	0.07 ± 0.03	0.06 ± 0.02

*Note*: Data are given as mean ± standard deviation unless otherwise noted.

Abbreviations: EEC, environmental exposure chamber; Expo, exposure; TOSS, Total Ocular Symptom Score; *P = 0.045, **P = 0.028 (Pearson correlations between Expo 2 and 4).

### 3.4 Kinetics of ocular symptoms after exposure in the EEC

**FIGURE 2:**
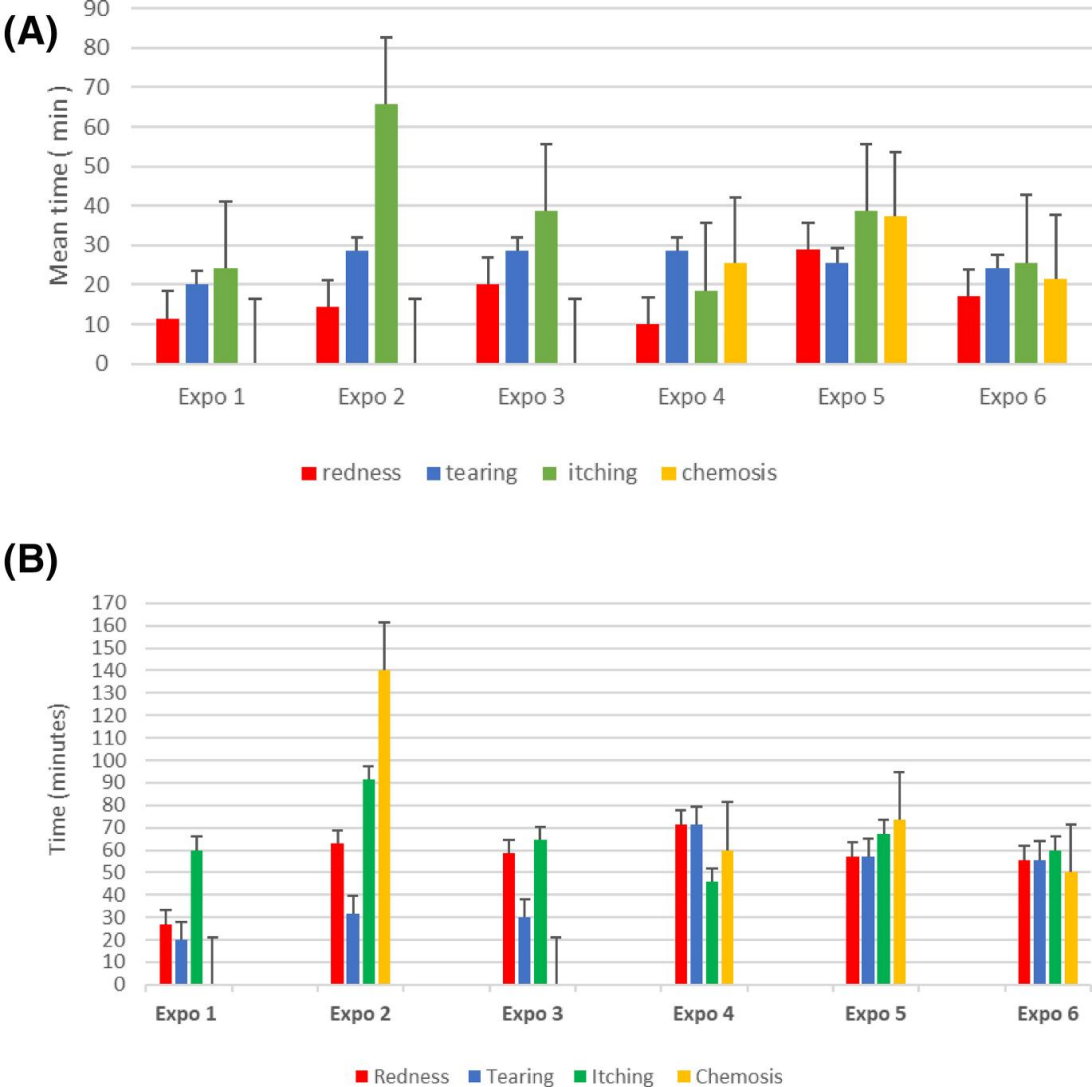
Step 2: Reproducibility. (A) Mean time to positive TOSS scoring after beginning EEC challenge (*n* = 7). Time in minutes; Error bars are presented as standard deviation (SD). (B) Time to reach maximal intensity of TOSS during exposures (Expos) in the EEC (*n *= 7). In Expo 2, only one patient experienced mild chemosis. Time in minutes; Error bars are presented as standard deviation (SD)

Time of onset and intensity of each symptom in the seven participants following step 2 are reported in Figure 2. During all six exposures, redness was the first symptom to appear, with a mean time of 16 ± 6.8 min, reaching maximum intensity in 55 ± 20.2 min. Tearing and itching appeared second, with a mean of 25 ± 3.4 min and 35 ± 16.9 min, respectively. Thus, reproducibility of redness, tearing, and itching occurred in 100% of individuals completing all six exposures. Chemosis was observed in Expo 4, 5, and 6 in six participants with a mean time of 28 ± 16.3 min after the patient entered the EEC. The maximum TOSS was 9 and occurred in three participants. No severe conjunctivitis was induced during exposure. All observed ocular reactions were considered mild and were controlled with a topical rescue treatment.

Nine participants (60% of the participants included) had a positive nasal response on the first two exposures (Expo 1 and Expo 2), confirmed by a positive TNSS ≥ 6 with a mean TNSS of 8.17 ± 1.47. This mean score was reached in 61 min. Moreover, patients who experienced a nasal response in the EEC did not have more severe conjunctival responses as compared with those without a TNSS ≥ 6. No significant difference in TOSS occurred between exposures**.** Five out of 16 participants developed an early asthma response during allergen exposure, with a mean decrease in FEV_1_ of 21.9% in 65 min on average (min 30 min; max 100 min). All early asthma reactions were treated by inhaled short‐acting beta‐2 agonist. Among participants presenting an early asthmatic response in the EEC, only one had a late asthmatic response, which was treated by oral corticosteroids and inhaled beta 2 agonist. No prolonged observation period was needed. No severe asthma reactions were observed during the study. No participants used the emergency kit provided during the test.

## 4 DISCUSSION

In the present study, when comparing ocular symptoms of participants exposed to birch pollen in the EEC compared to the reference CAC, we achieved the primary endpoint of 50% positivity in 16 participants during the first exposure. The following day, 75% of participants had a positive response. Furthermore, the airborne concentration of birch pollen inducing the response was very low, reaching a mean 60 ng/m^3^ of airborne Bet v 1 in the ALYATEC EEC. Different clinical studies have assessed the effect of allergen exposure in EECs on rhinoconjunctivitis, but very few have focused on allergic conjunctivitis. The time course of allergic signs and symptoms differed between the CAC and EEC sessions. In a previous study evaluating 13 participants with a history of ragweed allergy who underwent CAC and EEC exposure, the response time was different but the intensity of the maximal response was similar.^19^

The main inclusion criteria were a positive CAC, which is considered the gold standard for objectively evaluating conjunctival reactivity to a specific allergen at the mucosal surface. We chose a TOSS ≥ 5 during the CAC as the threshold for a conjunctival response according to European guidelines.^11^ This threshold has been demonstrated to allow a specificity and sensitivity of 100% and 90%, respectively, in mite allergic conjunctivitis.^20^ We used the same clinical positivity criteria for EEC exposure to birch. In our study, the mean TOSS was not significantly different after CAC and EEC exposure. The maximal TOSS was 9 in both the CAC and EEC. In contrast, the time necessary to obtain a positive response was significantly longer in the EEC than thae CAC. To the best of our knowledge, studies have not reported time between natural exposure and the occurrence of ocular symptoms. In daily practice, patients do not describe significant conjunctivitis symptoms in day‐to‐day life within 30 min after being exposed to birch pollen. When comparing patients with and without conjunctival positive scoring after EEC exposure, we observed no difference in terms of level of sensitization to birch pollens and also in terms of comorbidities. Consequently, the duration to obtain a significant clinical response to birch pollen in the EEC appeared to be closer to natural exposure than after a CAC. Moreover, the quantity of birch allergen inducing a conjunctival positive scoring was dramatically different between these two exposures. During CAC, positive responses were obtained with a mean cumulative dose of 980 ng of Bet v 1, whereas it was calculated to be 0.07 ng with the EEC. According to the HIALINE study, the amount of Bet v 1 per pollen grain can vary from 3.2 to 32 pg.^21^ Therefore, 980 ng of Bet v 1 corresponds to approximately 30,000 to 300,000 pollen grains. In the EEC, the amount of Bet v 1 inducing a positive response corresponds from 2 to 21 pollen grains. The literature assumed that participants allergic to pollen had symptoms as soon as the pollen grain threshold reached from 22 to 30 grains/m^3^ for grass pollens^22^ and 70 grains/m^3^ for birch pollen.^5^ Even though the manner of exposure is different, the results of natural and EEC exposure are similar, whereas challenge of the ocular surface through CAC exposes the individual to a much greater amount of allergen. Moreover, exposure in the CAC is performed through diluted allergen in NaCl solution instilled onto the ocular surface, whereas in EECs the allergens are nebulized in the air, which is a modality closer to naturel exposure.^11^ This triangular comparison enhances the clinical significance of the EEC challenge.

The conjunctival response in three‐fourths of participants during the second exposure in the EEC suggests that a priming effect occurs in terms of frequency of ocular signs and symptoms. However, we did not observe a difference in the severity of the TOSS between Expo 1 and Expo 2. The time inducing ocular responses was not significantly different between two consecutive exposures. Jacobs et al. suggested that no priming effect exists when exposures are performed on two consecutive days and that the conjunctivitis reaction that occurred on the second day may be a simple manifestation of a late phase reaction captured within a 24‐h period after exposure.^23^ Prior studies suggested that two, or even three, priming visits may be required to obtain high levels of symptoms.^24^, ^25^, ^26^ However, the priming effect leads to rapid onset of symptoms and signs rather than a greater allergic response.^27^

We observed reproducibility of ocular response frequency during all exposures. Reproducibility was assumed when challenging the ocular surface in the EEC for the time and the quantity of allergen inducing a clinical reaction.

Symptoms and signs induced in the EEC were comparable to those induced by CAC. Ocular redness was the first sign to appear in the EEC and lasted until the end of exposure. Its reproducibility was consistent across six allergen exposures. Our findings were in line with Jacobs et al.,^23^ who investigated phenotypes of allergic conjunctivitis. Other clinical symptoms of conjunctivitis, such as tearing and itching, occur rapidly. The kinetics of the appearance of the three main signs and symptoms is the same as in real life. Chemosis has also been associated with allergic conjunctivitis. When mild or moderate, chemosis requires slit‐lamp examination, which was used in both CAC and EEC exposure. Chemosis was not observed in our participants who submitted to CAC but was mild during EEC sessions. We observed good reproducibility of the kinetics of conjunctival symptoms: ocular redness, tearing, and ocular itching followed by chemosis. The latter can be considered a sign of severity, occurring when conjunctivitis was clearly present. The mild intensity of the chemosis when it was observed enhances the safety aspect of EEC exposure. The absence of severe loco‐regional symptoms, such as rhinitis, despite moderate to severe rhinitis reported in 50% of patients ‘medical history, is another argument that reinforces the safety of the technique. The threshold for a nasal response in EEC was defined as TNSS ≥ 6. Therefore, we considered it significant and reported only those who met this criterion. In this study, patients discharged EEC when TOSS ≥ 5. In such a situation, even though the symptoms of rhinitis appeared at the same time as those of conjunctivitis, only 60% achieved a TNSS ≥ 6. Conversely during CAC, none of the patients presented allergic rhinitis. To explain the higher occurrence of rhinitis in the EEC, a longer exposure to an aerosolized solution (droplets) is performed during EEC whereas it is a direct instillation Into the conjunctiva for CAC. Nevertheless, in five participants, we observed mild asthma symptoms during EEC exposure. This makes it possible to enroll mild asthma participants with allergic conjunctivitis when investigating ocular allergy. A control of spirometry parameters before, during, and after EEC challenge remains necessary. The difference between CAC and EEC types of exposure can account for discrepancies between side effects of both methods. Nevertheless, no serious adverse event occurred in patients after EEC exposure.

In our study, atopic status was evidenced by positive SPT to birch pollen and a high level of serum specific IgE. At inclusion, they reported birch pollen‐induced allergic conjunctivitis with or without rhinitis for at least 2 years with peak symptoms in March–April. All have a positive CAC at the inclusion. Nevertheless, 25% among them did not obtained a TOSS ≥ 5. These results could be explained by the difference of allergen amount during the two procedures. Allergen exposures in the EEC appear to be closer to natural exposure.

The limitation of this study is the small number of participants. However, we could demonstrate the clinical validity and good reproducibility of the method. The estimation of the amount of allergen deposition on the ocular surface could be discussed, but the calculation took into account the different physiological factors involved in the pathophysiology of conjunctivitis. Nevertheless, one can suggest that EEC exposures is closer to natural exposure than the CAC exposure.

## 5 CONCLUSION

We demonstrated that exposure to 60 ng/m^3^ of Bet v 1 in the ALYATEC EEC induces conjunctival positive scoring in more than 50% of participants with birch allergic conjunctivitis. Birch allergen exposures inducing early conjunctival responses were different than those identified with direct instillation during CAC, suggesting that EEC is closer to natural exposure during birch pollen season.

## CONFLICTS OF INTEREST

Alina Gherasim and Nathalie Domis are ALYATEC employees. Nathalie Domis is cofounder and ALYATEC employee. FdB is medical expert, cofounder, and shareholder of ALYATEC. Frederic de Blay reports grants from STALLERGENES GREER, grants from CHIESI and REGENERON, and personal fees from ALK ABELLO, MUNDIPHARMA, NOVARTIS, and is a member of the Board at STALLERGENES GREER, NOVARTIS, ALK ABELLO, MUNDIPHARMA, MEDAPHARMA, BOEHRINGER, ASTRAZENECA, and CALOR. Xavier Siomboing and Jean‐Luc Fauquert: none.
